# Concordance, Correlation, and Clinical Impact of Standardized PD-L1 and TIL Scoring in SCCHN

**DOI:** 10.3390/cancers14102431

**Published:** 2022-05-14

**Authors:** Stijn Jeroen De Keukeleire, Tijl Vermassen, Philippe Deron, Wouter Huvenne, Fréderic Duprez, David Creytens, Jo Van Dorpe, Liesbeth Ferdinande, Sylvie Rottey

**Affiliations:** 1 Department of Medical Oncology, University Hospital Ghent, 9000 Ghent, Belgium; tijl.vermassen@uzgent.be (T.V.); sylvie.rottey@ugent.be (S.R.); 2 Department of Internal Medicine, University Hospital Brussels, 1090 Jette, Belgium; 3 Drug Research Unit Ghent, Ghent University Hospital, 9000 Ghent, Belgium; 4 Cancer Research Institute Ghent (CRIG), 9000 Ghent, Belgium; 5 Department of Head and Neck Surgery, Ghent University Hospital, 9000 Ghent, Belgium; philippe.deron@uzgent.be (P.D.); wouter.huvenne@uzgent.be (W.H.); 6 Department of Radiation Oncology, Ghent University Hospital, 9000 Ghent, Belgium; frederic.duprez@uzgent.be; 7 Department of Pathology, Ghent University Hospital, 9000 Ghent, Belgium; david.creytens@uzgent.be (D.C.); jo.vandorpe@uzgent.be (J.V.D.); liesbeth.ferdinande@ugent.be (L.F.)

**Keywords:** squamous cell carcinoma of head and neck, TILs, PD-L1, CPS, spatiotemporal heterogeneity, concordance, prognosis

## Abstract

**Simple Summary:**

In patients with relapsed or metastasized squamous cell cancer of the head and neck (R/M SCCHN), the PD-L1 Combined Positive Score (CPS) is currently the only predictive biomarker for treatment with anti-PD-1 agents. However, ambiguous results have been determined regarding the overall response rates of immunotherapeutic agents based on PD-L1 status, which may be partially attributed to spatiotemporal heterogeneity. Furthermore, tumor-infiltrating lymphocytes (TILs) have proven to be of significant prognostic value, yet lack of a standardized method for quantification impedes their integration into the current armamentarium of biomarkers in SCCHN. In this paper, concordance of PD-L1 CPS and stromal TILs was investigated in different paired samples of SCCHN subtypes. The results were then linked to well-known clinicopathological variables and prognosis.

**Abstract:**

Background: The clinical significance of tumor-infiltrating lymphocytes (TILs) and programmed cell death-ligand 1 (PD-L1) expression has been thoroughly researched in squamous cell carcinoma of the head and neck (SCCHN). To address the impact of intra- and intertumoral heterogeneity in these biomarkers, we explored the concordance of PD-L1 combined positive score (CPS) and stromal TILs in different paired tissue sample types, while evaluating their internal relationship and prognostic impact. Methods: A total of 165 tissue blocks from 80 SCCHN patients were reviewed for TILs and PD-L1 CPS. Concordance between paired tissue samples was evaluated, and their association with several clinicopathological variables, overall survival (OS), and disease-free survival (DFS) was determined. Results: Biopsies and paired resection material were severely discordant in 39% and 34% of samples for CPS and TIL count, respectively, of which CPS was underscored in 27% of biopsies. In paired primary tumor–metastatic lesions, the disagreement was lower for CPS (19%) but not for TIL count (44%). PD-L1 CPS was correlated with prolonged OS when calculated from tissue acquirement, while extended OS and DFS were observed for high TIL density. Conclusion: Intertumoral and, especially, intratumoral heterogeneity were confounding factors when determining PD-L1 CPS and TIL count on paired tissue samples, indicating the increasing necessity of assessing both biomarkers on representative tissue material. Although TILs hold valuable prognostic information in SCCHN, the robustness of PD-L1 as a biomarker in SCCHN remains ambiguous.

## 1. Introduction

Squamous cell carcinoma of the head and neck (SCCHN) is a heterogeneous disease characterized by a wide variety of genetic alterations, residing in a largely immunosuppressive environment. Patients with SCCHN have a 5-year overall survival (OS) probability of 65% when considered across all patients’ ages and stages. However, the majority will present with locally advanced or recurrent/metastatic (R/M) SCCHN, carrying poorer prognosis [1,2].

The blockade of the programmed cell death-1 (PD-1)/programmed cell death-ligand 1 (PD-L1) axis by means of ICI is considered a landmark event in several malignancies, including SCCHN, resulting in clear survival benefit for patients [2,3,4,5]. In Europe, pembrolizumab-based therapy is implemented as a first-line therapy in patients with R/M SCCHN, be it in monotherapy or in combination with platin-based chemotherapy. To increase the response rate to pembrolizumab, PD-L1 immunohistochemistry (IHC) was brought in as a predictive biomarker. Tumors with a combined positive score (CPS), i.e., the combined scoring of PD-L1-expressing tumor cells (TCs) and all immune cells (ICs), of ≥20, and to a lesser extent ≥1, have an increased objective response rate and post-therapy survival [6,7,8,9,10].

Next to the CPS, the immune infiltrate, of which TILs are a crucial component, has been proven to harbor significant prognostic value. To this end, the International Immuno-Oncology Biomarker Working Group (IIBWG) has introduced a standardized method for tumor-infiltrating lymphocyte (TIL) quantification in clinical practice, encompassing several types of solid malignancies, including SCCHN [11,12]. Recent evidence from our research group demonstrated that using this standardized scoring method, high TIL count is an independent prognostic factor in oropharyngeal squamous cell carcinoma (OPSCC) [13]. Although the importance of TILs has been presented in literature, this tissue biomarker is yet to be implemented in daily clinical practice.

Although it has been recognized that both CPS and TILs play an important role in the tumor microenvironment of SCCHN, further investigation is still needed. Several reports have described spatiotemporal heterogeneity of PD-L1 expression in various solid carcinomas, with subsequent impact on the treatment choice and outcome [14,15,16,17,18,19,20,21,22]. This suggests that the PD-L1 CPS results can vary depending on the size and origin of the tissue sample that is available for immunohistochemical testing (biopsy/resection, specimen/metastasis). Moreover, limited to no information is available on the internal relationship between TILs and the CPS, and on how this could affect the scores.

In the current section, we investigated the heterogeneity of PD-L1 CPS on three levels: (1) intratumoral; between different sample types (biopsies vs. resection of primary tumors), (2) intertumoral; between different tissue types (primary tumor vs. lymph node and distant metastases), and (3) temporal; as time progresses during the disease course. Next, the correlation between TILs and CPS is defined, as well as their correlations with several well-known clinicopathological variables. Lastly, the clinical benefit of TIL scoring and CPS as prognostic biomarkers is determined.

## 2. Materials and Methods

### 2.1. Population and Sample Selection

Eighty patients with histologically confirmed SCCHN at any primary site (oral cavity, oropharynx, hypopharynx, or larynx) were included when ≥2 different tissue samples were available. Tissue specimens were retrieved between January 2017 and December 2019 at the department of Pathology, University Hospital, Ghent. Nine patients were IHC p16^+^, of which seven were OPSCC. Patient characteristics are shown in Table 1. According to current recommendations in the literature, IHC PD-L1 was performed on archival blocks that did not exceed three years of age [23]. Cytological material as well as formalin-fixed paraffin-embedded blocks that underwent decalcification, were excluded [24,25]. Tissue material originated from primary tumors, as biopsies or resected material, and metastatic lesions. A total of 163 tissue blocks were available: 58 biopsy specimens, 63 resected specimens, 36 lymph node metastases, and 6 distant metastases. Paired tissue samples were available between biopsy and resection material (*n* = 44), resection material and lymph node metastasis (*n* = 27), biopsy and lymph node metastasis (*n* = 18), or any material from primary tumor and any metastatic lesion (*n* = 36). For 11 patients, we retrieved paired biopsy, resection, and lymph node specimens. The study was approved by the ethics committee of the University Hospital, Ghent.

### 2.2. Evaluation of the Tumor Microenvironment

**Figure 1:**
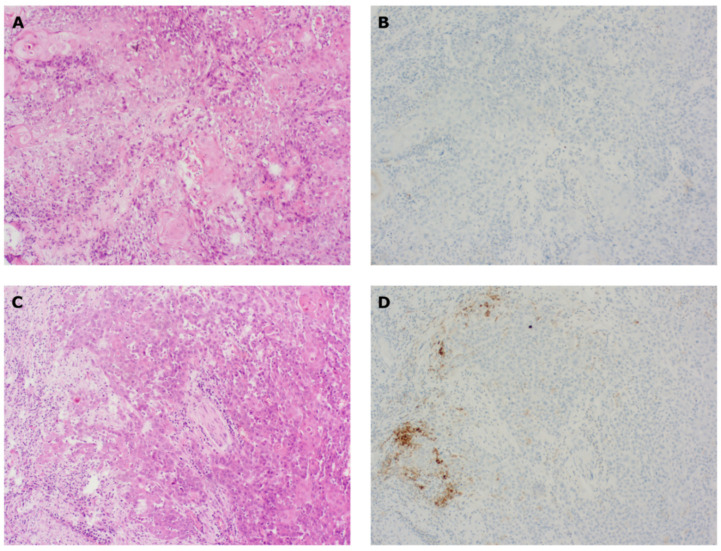
Effect of insufficient stroma present on PD-L1 CPS in paired specimens. All samples were derived from the same SCCHN patient and stained with HE (**A**,**C**) or PD-L1 (**B**,**D**). (**A**,**B**) Biopsy from the primary tumor with insufficient tumor stroma and absent PD-L1 staining (CPS = 0). (**C**,**D**) Resection specimen from the primary tumor with adequate amount of tumor stroma. PD-L1 staining was absent on TCs but partially present on ICs (CPS = 5). CPS, combined positive score; HE, hematoxylin–eosin; IC, immune cell; IHC, immunohistochemistry; PD-L1, programmed cell death-ligand 1; SCCHN, squamous cell cancer in head and neck; TC, tumor cell.

Tumor slides were evaluated for PD-L1 expression (CPS) and the presence of TILs. All slides were evaluated by two senior pathologists and one trained PhD fellow. Tissue slides were additionally checked for possible confounding factors, such as the presence of inflammatory sites (erosion, ulceration, and/or necrotic debris) and insufficient amount of tumoral stroma (defined as a ratio of tumoral stroma/TCs of <10%) (Figure 1).

#### 2.2.1. PD-L1 IHC

Paraffin sections were immunohistochemically stained for PD-L1 using the 22C3 antibody (Dako/Agilent, Santa Clara, CA, USA) on the Ventana (Roche, Basel, Switzerland) staining platform using the protocol described by Neuman et al. [26]. Blocks were sectioned at a thickness of 3 μm and stained on positively charged glass slides. The CPS is described as the number of positive tumor cells, lymphocytes, and macrophages, divided by the total number of viable tumor cells multiplied by 100. CPS was considered valid when >100 viable TCs were present. PD-L1+ cells were scored when (partial) membranous staining was noted in the case of TCs, while ICs (lymphocytes and macrophages) were allowed membranous or cytoplasmic staining. In the case of lymph nodes, PD-L1+ ICs were included if within 0.5 mm of the TCs [27,28]. CPS was further categorized based on the median CPS of all three observers. The threshold of PD-L1 positivity was set at 1 (CPS < 1 vs. CPS ≥ 1). PD-L1+ tumors were further distinguished in moderate (1–19) and high (≥20) CPS. Automated image analysis or computational histological assessment have gained interest in the field of IHC markers, and although this may improve the accuracy and reproducibility of PD-L1 assessment in tumor specimens, tissue slides were all manually scored by all three observers in this study [29,30].

#### 2.2.2. TIL Scoring

TILs were evaluated according to the protocol of the IIBWG. For the extensive methodology, we refer the reader to the article from our research group [13]. Briefly, HE-stained sections containing invasive SCC were scored for mononuclear inflammatory cells, including lymphocytes, plasma cells, and macrophages in stromal tissue, while granulocytes were excluded from evaluation. We calculated the percentage of TILs in the area of stromal tissue occupied by mononuclear inflammatory cells over the total stromal area. TILs were reported as a continuous variable (%) and were further categorized based on the median TILs of all three observers.

### 2.3. Statistics

TILs and CPS were assessed as both continuous and categorical variables. Dichotomization of TILs was determined via Receiver operating curve analysis, using ‘death’ as the classification variable. Interobserver variability was assessed using the intraclass correlation coefficient (ICC) or Fleiss multirater κ for continuous and categorical variables, respectively. Concordance of CPS and TILs for paired tissue samples was assessed by determining the difference between paired tissue samples using a Wilcoxon test (two categories) and Friedman test (three categories). Furthermore, concordance for CPS and TILs as categorical variables was assessed using chi-square (χ^2^) or Fisher’s exact test. Temporal PD-L1 and TILs heterogeneity was assessed by calculating the correlation (Pearson’s r) between change in CPS (ΔCPS) and change in TIL count (ΔTILs) of paired samples, and Δtime. Next, the associations between CPS, TILs, and several well-known clinicopathological variables were determined by Pearson’s correlation, and Kruskal–Wallis and χ^2^ tests. The preferred tissue to be used in the survival analysis was selected based on the concordance results, tissue availability, criteria from recommendations in the literature, and our own experience. Herein, resection specimens were considered superior over biopsies in the case of primary tumors and, when available, metastatic specimens were preferred as they delivered the most recent PD-L1 status. Lymph node metastases were not the preferred sample types due to the possible impact of the abundant presence of non-TIL lymphocytes on PD-L1 and TIL scoring. As a result, the following tissue blocks were used in the survival analysis: 62 resections, 10 biopsies, 4 distant metastases, and 2 lymph nodes. OS was calculated as: (1) from time of (biopsy-proven) diagnosis, and (2) from time of tissue acquirement until date of death by any cause or final follow-up. Disease-free survival (DFS) was calculated from time of tissue acquirement until date of relapse, death by any cause, or final follow-up. Four patients did not qualify for DFS analysis as metastatic disease was observed at time of tissue acquirement. The survival curves were plotted using the Kaplan–Meier method. Univariate HR of each baseline and clinical parameter of OS and DFS was determined by a log-rank (Mantel–Cox) test. Multivariate analyses were conducted using the Cox regression model on the variables that were statistically significant upon univariate analysis. All applied tests were calculated as two-sided with a statistical significance level of 0.05.

## 3. Results

### 3.1. PD-L1 CPS and TIL Scores

Median CPS was highest for lymph node metastases, followed by primary tumor resection material, distant metastases, and lastly, primary tumor biopsies. PD-L1 positivity (CPS ≥ 1) was noticed in 69%, 86%, 86%, and 50% of cases for primary tumor biopsies, primary tumor resection, lymph node metastases, and distant metastases, respectively. Otherwise, TIL score was highest in both primary tumor resection material and lymph node metastases, followed by primary tumor biopsies, whereas distant metastases showed the lowest TIL count. The median TIL score was 20% (range 0–90%) for all samples. The cut-off point for further analysis was set at 20% to discriminate between high and low TIL density, which was determined post hoc (Table 2).

For the evaluation of all tumor tissue, irrespective of the tissue origin, the interobserver variability in terms of ICC showed excellent concordance for CPS assessed as a continuous variable (*n* = 161, ICC_absolute agreement_ = 0.908, ICC_consistency_ = 0.910) as well as for TIL count (*n* = 137, ICC_absolute agreement_ = 0.747, ICC_consistency_ = 0.767). When evaluating the inter-rater concordance in terms of categorical variables, substantial agreement was reached between observers for CPS as a dichotomous variable (CPS < 1 vs. CPS ≥ 1, κ = 0.753, *p* < 0.001) and for CPS as a trichotomous variable (CPS < 1 vs. CPS 1–19 vs. CPS ≥ 20, κ = 0.694, *p* < 0.001), whereas only moderate agreement was achieved for TIL count (κ = 0.406, *p* < 0.001).

A lack of sufficient stroma was observed in 28/58 biopsies, and was associated with lower median CPS (3.5 vs. 1.0, *p* = 0.0399) but not lower number of TILs (15.0 vs. 16.5, *p* = 0.7408). For resection material, stromal insufficiency was observed in 9/63 specimens, and was not associated with alterations in CPS (5.0 vs. 5.0, *p* = 0.5772), although a trend towards significance was observed for TILs (20.0 vs. 10.0, *p* = 0.0828). No *p* value was obtained for (lymph node or distant) metastases due to the restricted sample size.

### 3.2. Tissue Concordance

#### 3.2.1. CPS

**Figure 2:**
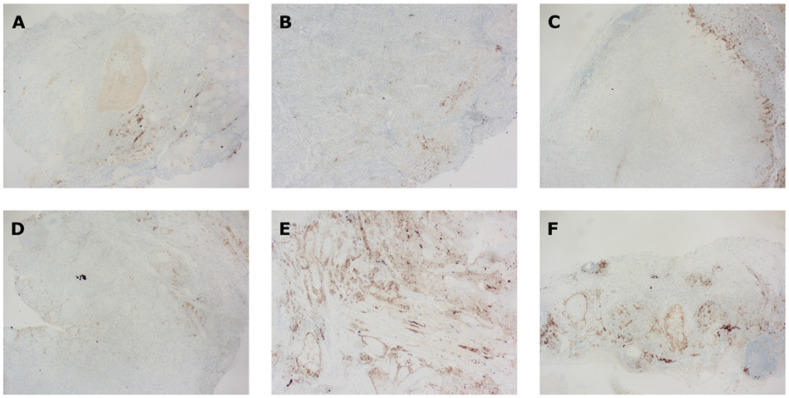
PD-L1 IHC of paired SCCHN tissue samples. Paired specimens of two patients (**A**–**C** vs. **D**–**F**) are shown: primary tumor biopsies (**A**,**D**), resected primary tumors (**B**,**E**) and lymph node resections (**C**,**F**). In the upper row (**A**–**C**), all paired tissue samples had concordant PD-L1 CPS between 1 and 5. In the lower row, discordant PD-L1 CPS was noticed with biopsy CPS = 1–5 (**D**) vs. resection and lymph node CPS ≥ 20 (**E**,**F**). CPS, combined positive score; IHC, immunohistochemistry; PD-L1, programmed cell death-ligand 1; SCCHN, squamous cell cancer in head and neck.

A significant difference in CPS was observed between biopsy and resection material (intratumoral heterogeneity, *p* = 0.0085), between resection and lymph node material (intertumoral heterogeneity, *p* = 0.0495), and between biopsy and lymph node material (intra- and intertumoral heterogeneity, *p* = 0.0076). Interestingly, no difference was seen when comparing primary tumor tissue with metastatic tissue (*p* = 0.3669). For patients with available paired biopsy, resection, and lymph node material (*n* = 11), a significantly lower CPS for biopsies was observed (*p* = 0.0017; Table S1; Figures S1 and S2). A comparison between paired tissue samples (biopsy vs. resection vs. lymph node) for a concordant and discordant PD-L1 CPS is illustrated in Figure 2. Despite the objectified differences in CPS shown for paired tissues, the correlation of CPS between paired tissue specimens was determined. Here, it was clear that a positive correlation could be observed between biopsy and resection specimens, between resection and lymph node specimens, and between primary tumor material and metastatic lesions. Interestingly, no correlation could be found between biopsy and lymph node specimens (Table S2; Figure S3).

When using CPS as a dichotomous variable, 27/44 (61%) paired biopsy–resection samples had concordant PD-L1 status. Five biopsies (11%) were considered to be PD-L1+, although the corresponding resection sample was considered PD-L1−. Conversely, 12 biopsies (27%) were PD-L1−, whereas the paired resection specimens were found to be PD-L1+. Consequently, no association was found between CPS of paired resection and biopsy material (*p* = 1.000). When using CPS as a trichotomous variable (CPS < 1, CPS 1–19, CPS ≥ 20), 20/44 (45%) of samples had a fully concordant CPS, showing no association between paired resection material and biopsies (*p* = 0.1986; Table 3A and Table S3A). Likewise, it was noticed that biopsy material underestimated the CPS when comparing corresponding lymph node material. Here, 5/18 biopsies were considered to be PD-L1−, while the paired lymph node was PD-L1+ (*p* = 0.0686; Table 3B and Table S3B). Next, an association was observed between resection material and lymph nodes for CPS as a trichotomous variable (*p* = 0.0016). Considering the dichotomized variable, 24/27 (89%) of samples were attributed similar scores, with only three lymph nodes being PD-L1−, whereas the paired resection specimens were all PD-L1+ (*p* = 0.1481, Table 3C and Table S3C). An association was also seen between primary tumor material and metastatic tissue for CPS as a trichotomous variable (*p* = 0.0082), but not as a dichotomous variable (*p* = 0.1858). In the latter, 4/36 and 3/36 of metastatic samples were over- and underestimated, respectively, with regard to the primary tumor material (Table 3D and Table S3D). Lastly, 11 patients had paired specimens from primary tumor biopsy, resection, and lymph node material. As a dichotomous variable, CPS was concordant in 6/11 (55%) of paired samples. Moreover, paired samples reached poor agreement when applying CPS as a trichotomous variable, with 3/11 (27%) samples having a fully concordant CPS (Table S4).

#### 3.2.2. TIL Count

Comparable to CPS, the concordance of TIL scoring was assessed between different tissue samples. No significant discordance was observed among paired samples (Table S5; Figures S2 and S4). Furthermore, a positive correlation could only be observed between biopsy and resection specimens, whereas this was absent between resection and lymph node specimens, biopsy and lymph node specimens, and primary tumor material and metastatic lesions (Table S6; Figure S5). When using TILs as a dichotomous variable (TILs < 20% vs. TILs ≥ 20%), an association was found between paired biopsy and resection samples (*p* = 0.0251), with concordant scoring in 27/41 samples (66%). Comparable to the CPS, the majority of discordant samples were underscored in biopsy specimens (11/41, 27%; Table 3A). No associations were observed in the comparison between paired biopsy and lymph node samples (*p* = 1.0000; Table 3B), paired resection and lymph node samples (*p* = 1.0000; Table 3C), or between paired primary tumor and metastatic samples (*p* = 0.6882; Table 3D). Among patients with paired specimens from primary tumor biopsy, resection, and lymph node samples, TIL concordance was seen in 4/11 samples (36%; Table S7).

### 3.3. Temporal Heterogeneity

Temporal heterogeneity and its effects on CPS and TILs were assessed in all paired specimens. To our knowledge, no standardized evaluation method for assessing temporal heterogeneity has thus far been described in literature. We investigated ΔCPS and ΔTILs over time between paired tissue samples as the correlation between ΔCPS/ΔTILs and Δtime for all specimen types using both linear and exponential models. No significant correlations were found. All correlations are given in Table S8.

### 3.4. Correlation between CPS, TILs, and Clinicopathological Parameters

As continuous variables, several correlations could be found between CPS and TILs. A positive correlation between CPS and TILs was present between all samples, irrespective of their origin or the implemented correlation model (Table S9, Figure S6). Further stratification according to tissue sample type indicated that the observed correlation was mostly valid for resection specimens, less so for distant metastases, and not at all for biopsy and lymph node specimens. The correlation between CPS and TILs held up for tissue specimens selected for the survival analysis (Table S9). As categorical variables, on the other hand, CPS and TILs were not associated with each other, neither for CPS as a trichotomous variable (*p* = 0.1934) or as a dichotomous variable (*p* = 0.1242).

Secondly, the association between CPS/TILs and several well-known clinicopathological parameters was evaluated (Table 4). Notably, patients who had smoked frequently (≥20 pack-years) had a higher CPS compared to patients with less pack-years (PD-L1 positivity of 87% vs. 76%, respectively). In addition, patients with moderately or poorly differentiated tumors also had more PD-L1+ tumors (86% and 96%, respectively) in comparison with patients with tumors differentiated as good or basaloid (57% and 60%, respectively). This was also true for CPS as a continuous variable (Figure S7). None of the clinicopathological parameters could be linked to the TIL status. Tumors located in the oral cavity and oropharynx tended to have augmented TIL counts, but this was not statistically significant (*p* = 0.1328; Figure S7).

### 3.5. Effect of CPS and TILs on Survival Outcome

#### 3.5.1. DFS

Patients with only a metastatic lesion available (*n* = 4) were excluded from the DFS analysis. Forty-four patients (58%) experienced any event (relapse, disease progression, or death). In contrast to OS, T stage did not have any influence on the duration of DFS since time of therapy, nor did any of the other clinicopathological parameters (Table S10). Next, trichotomous CPS showed no association with DFS outcome (median DFS of 1.5 years, 2.7 years, and not reached, for CPS < 1, CPS 1–19, and CPS ≥ 20, respectively, *p* = 0.6426; Figure S8A). Comparably, dichotomous CPS showed no prognostic value for DFS (median DFS 1.5 years vs. 2.7 years, hazard ratio (HR) CPS ≥ 1 = 0.68 (0.27–1.70), *p* = 0.4070; Figure S8B). The only variable linked to DFS was TIL count. Similar to OS, having a high TIL count was associated with a 55% decrease in risk of any disease event (median DFS 1.4 years vs. 4.0 years, HR TILs ≥ 20% = 0.45 (0.24–0.84), *p* = 0.0126; Figure S8C). An overview of the univariate DFS outcome is given in Table S10.

#### 3.5.2. OS since Time of Diagnosis

**Figure 3:**
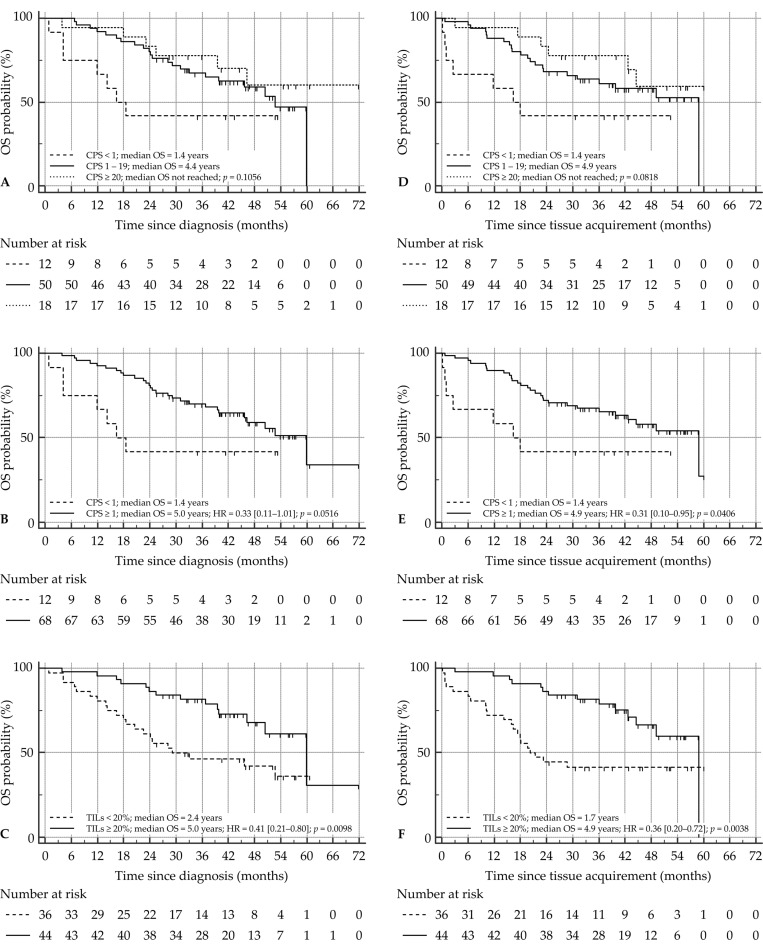
Effect of CPS and TIL count on OS in SCCHN patients. *x*-axis depicts survival time (in months), *y*-axis depicts the cumulative OS probability. OS since diagnosis is depicted for (**A**) trichotomous CPS, (**B**) dichotomous CPS, and (**C**) TILs. OS since time of tissue acquirement is depicted for (**D**) trichotomous CPS, (**E**) dichotomous CPS, and (**F**) TILs. Number at risk for each group is indicated beneath each OS curve. CPS, combined positive score; HR, hazard ratio; OS, overall survival; SCCHN, squamous cell carcinoma of the head and neck; TIL, tumor-infiltrating lymphocyte.

**Figure 4:**
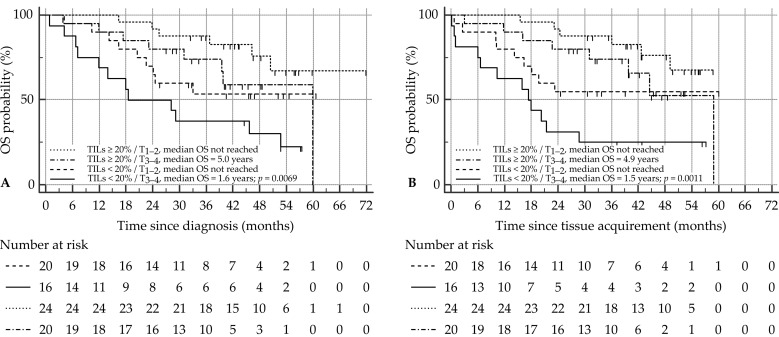
Effect of combination of T stage and TIL count on OS in SCCHN patients. *x*-axis depicts survival time (in months), *y*-axis depicts the cumulative OS probability. Survival curves are depicted for (**A**) OS since diagnosis and (**B**) OS since time of tissue acquirement. Number at risk for each group is indicated beneath each OS curve. HR, hazard ratio; OS, overall survival; SCCHN, squamous cell carcinoma of the head and neck; TILs, tumor-infiltrating lymphocytes.

A total of 35 patients (44%) had died at time of analysis. For all clinicopathological variables, only a low T stage (T1–2) was associated with prolonged OS in this SCCHN patient cohort (*p* = 0.0310). None of the other variables showed prognostic properties in the case of OS outcome, although alcohol abuse and smoking history both showed a trend towards significance (Table S11). Patients with high PD-L1 expression tended to live longer (median OS of 1.4 years, 4.4 years, and not reached, for CPS < 1, CPS 1–19, and CPS ≥ 20, respectively) although this was not statistically significant (*p* = 0.1056; Figure 3A), nor was it significant when CPS was implemented as a dichotomous variable (median OS 1.4 years vs. 5.0 years, HR CPS ≥ 1 = 0.33 (0.11–1.01), *p* = 0.0516; Figure 3B). TILs, on the other hand, proved to be a significant prognosticator for OS, with patients possessing a high TIL count (≥20%) surviving twice as long compared to those with a low TIL count (<20%; median OS 2.4 years vs. 5.0 years, HR TILs ≥ 20% = 0.41 (0.21–0.80), *p* = 0.0098; Figure 3C). Multivariate analysis showed that both T stage and TILs are independent prognosticators for OS (Table S11). Combining these together into one prognostic marker proved significant, with T1–2 tumors possessing a high TIL count having the best outcome (*p* = 0.0069; Figure 4A).

#### 3.5.3. OS since Time of Tissue Acquirement

A major time difference may exist between the time of diagnosis and the moment of tissue acquirement and analysis (as in the case of lymph node specimens or metastatic lesions), which may hamper OS analysis. Therefore, OS was also calculated from time of tissue acquirement. In the univariate analysis, the trichotomous CPS again only showed a trend towards significance for OS outcome (median OS of 1.4 years, 4.9 years, and not reached, for CPS < 1, CPS 1–19, and CPS ≥ 20, respectively, *p* = 0.0818; Figure 3D). Using the dichotomous CPS, on the other hand, high CPS was indicative of an improved OS (median OS 1.4 years vs. 4.9 years, HR CPS ≥ 1 = 0.31 (0.10–0.95), *p* = 0.0406; Figure 3E). T stage and TILs remained significant prognosticators for OS (Table S12; Figure 3F). Moreover, CPS was not withheld in the multivariate analysis, alongside T stage and TIL count (Table S12). Similar to OS since time of diagnosis, the combination of T stage and TIL count was significant for OS outcome (*p* = 0.0011; Figure 4B).

## 4. Discussion

In this heterogeneous population of SCCHN, we assessed both PD-L1 expression and TILs, according to a standardized methodology, on several tissue specimens. Our first goal was to elucidate the concordance of PD-L1 CPS and TIL quantification between different SCCHN tissue sample types. Next, we evaluated their internal relationship and association with routinely used clinicopathological variables. Lastly, we determined the prognostic properties of both parameters in terms of survival outcome, especially OS and DFS.

Firstly, we demonstrated that intratumoral heterogeneity can be a major confounding factor when assessing PD-L1 status in SCCHN: a substantial amount of primary tumor biopsies were found to have discordant CPS compared to their paired resection specimens. Indeed, biopsy samples had a higher risk of being underscored (27%), and to a lesser extent, overscored (11%). In clinical practice, this would mean that treatment decisions would be different in two out of five patients (38%) when performing PD-L1 IHC on biopsy samples instead of on larger resection specimens. One out of four patients will be excluded from PD-L1-targeted therapy due to this impact of heterogeneous PD-L1 expression, while they would have received the therapy if the test was executed on resection material. These results are similar to previous reports: Rasmussen et al. [14] evaluated concordance of PD-L1 expression between paired biopsy and resection material. After completion of surgery, multiple core biopsies were performed on the ‘en bloc’ resected tumor. Indeed, around half of the biopsies were falsely assigned a PD-L1− status (CPS cut-off = 1) [14]. Next, Paintal et al. [24] found the PD-L1 status of biopsies to be discordant in 30% compared to paired resection material [24]. Comparable results were found for the intratumoral concordance of TIL assessment, where the evaluation of the biopsy specimens, in the case of discordance, mostly resulted in a lower number of high TIL samples (27%) compared to resection specimens.

Additionally, we discovered that a lack of tumoral stroma (in our study defined as a ratio of tumoral stroma/TCs of <10%) negatively affects the CPS in biopsies. When evaluating PD-L1 according to the CPS assay, ICs contribute to the final PD-L1 status, especially in samples with a low amount of PD-L1+ TCs. Lack of stroma may therefore preclude adequate scoring of ICs and enhance the risk of underscoring CPS in biopsy samples. Indeed, Paintal et al. [24] reported that the discordance between biopsies and resection samples was mainly noticed for PD-L1-expressing ICs, while TC staining was mostly similar in paired samples [24]. Therefore, a sufficient amount of TCs (>100), together with an adequate amount of tumoral stroma, should count as essential prerequisites to perform PD-L1 CPS on tissue samples, particularly in biopsy material. Interestingly, the lack of tumoral stroma did not affect the TIL count.

As for intertumoral heterogeneity of PD-L1 expression, we observed significant discordance between primary tumors (independent of specimen type) and paired metastatic lesions, reaching up to 19% for dichotomous, and 33% for trichotomous CPS. For the TIL count, this was even worse, with a discordance of up to 44%. In SCCHN, only scarce data are available regarding PD-L1 concordance in different tumor sample types. Previous reports from other cancer types have shown PD-L1 CPS in lymph nodes to be overscored due to a higher prevalence of PD-L1+ ICs residing in pre-existing lymphoid tissue [15,16,17,18]. In our cohort, the observed mean CPS was indeed highest in lymph nodes. However, lymph nodes and resected primary tumors were distributed equally when categorizing CPS as a di- or trichotomous variable. In line with our data, Straub et al. [19] reported that primary tumors of patients with OSCC had discordant PD-L1 status (expressed on TCs only) in 28% of paired cervical lymph node metastases [19]. Concerning distant metastases, Okada et al. [20] analyzed PD-L1 expression in primary tumors and paired resected pulmonary metastases, revealing significant discordance in PD-L1-expressing TCs. PD-L1 status conversion was shown in 16/26 (62%) paired samples: 10 metastatic lesions were PD-L1+ though were identified as PD-L1− on paired primary tumor resections, and the reverse occurred in six metastatic lesions [20]. A recently published meta-analysis confirmed that metastatic lesions in SCCHN have the highest rate of PD-L1 conversion of all solid tumors [21]. In our study cohort, three out of four distant metastases indeed showed PD-L1 conversion, yet the small sample size of this group limits the importance of this finding. The abovementioned studies predominantly reported PD-L1 discordances assessed on TCs only, while limited consideration is given to PD-L1+ ICs. Scognamiglio et al. [31] demonstrated poor concordance (56%) for PD-L1-expressing TCs in paired primary tumors and cervical lymph node metastases, although when adjusting for PD-L1-expressing ICs, agreement significantly improved (77%) [31].

Assessing the temporal heterogeneity of PD-L1 is a complicated procedure. PD-L1 expression is considered a time-dependent biomarker prone to longitudinal fluctuations. However, it cannot be ruled out that the current findings in the literature regarding temporal heterogeneity may be partially explained by PD-L1 discordance due to intra- and/or intertumoral heterogeneity, especially as biopsy tumoral material is the most common tissue sample used for diagnosing recurrence in daily oncological practice and for performing subsequent additional testing, such as PD-L1 CPS. We explored temporal heterogeneity by correlating the variables time and change in CPS (∆CPS), using both linear and exponential models. No significant correlation was observed for any of the sample types, irrespective of the implemented model, indicating the high variability associated with temporal heterogeneity. Ideally, temporal heterogeneity should be prospectively assessed at multiple sequential time points using high-quality tissue samples to determine the variations in PD-L1 CPS. However, this would be an unfeasible study protocol in this setting.

As a second objective, an investigation into the mutual association between the CPS and TIL count, as well as the association of each of these separate pathological markers with other clinicopathological biomarkers, was attempted. A positive but modest correlation between PD-L1 CPS and TILs was observed for both linear and exponential models. This was almost exclusively found among resection specimens, whereas no correlation could be found for biopsy or lymph node samples. Other studies have also reported PD-L1 expression to be higher in areas with increased immune infiltration. As the CPS assay allows the scoring of PD-L1-expressing ICs, this can be regarded as an evident relationship [32,33]. However, we detected a significant group of cases that were PD-L1+ (CPS ≥ 1) with low TIL density, and vice versa. Consequently, theories correlating high PD-L1 expression to a pro-tumorigenic immunosuppressive environment, or associating high TIL density as a prerequisite of anti-tumor immunity, seem oversimplified [34,35]. Scognamiglio et al. [31] reported that the PD-L1 expression in SCCHN may be distinguished as two phenotypes. Firstly, the induced expression mediated by adaptive immunity and attributed to IFNγ signaling between TCs and ICs; PD-L1 expression is induced and dependent on the immunogenicity, the tumor mutational burden, and/or local TIL density within the tumor. Alternatively, there is the constitutive expression: PD-L1 expression is not correlated with immunogenicity or TIL density, but exhibits the innate, or constitutive, expression of PD-L1 of TCs [31]. This is an interesting assumption that could partially explain the different patterns between TIL density and PD-L1 expression that were noticed in several samples, especially in the lymph node samples (Figure S9).

Regarding clinicopathological variables, we found that patients with absent to moderate tobacco use were associated with a higher CPS (≥20). Furthermore, it seems that those with restricted alcohol consumption tend to have tumors with increased TILs (≥20%). These well-known behavioral risk factors may exhibit a potential impact on the shaping of the tumor microenvironment in SCCHN, which has been similarly described in previous publications [36,37]. We reported PD-L1+ tumors to be highly represented in moderately and poorly differentiated tumors compared to other phenotypes. Indeed, expression of PD-L1 has been associated with histopathological grade in various malignancies [38]. Similar observations have been reported in patients with NSCLC, and in one study, for patients with OSCC, in which low PD-L1 expression was correlated to well-differentiated tumors (<4 cm in size) [39,40,41]. These findings suggest that PD-L1 expression could co-function as a mediator of tumoral progression. Concerning TILs, no other significant association was displayed in regard to histological grade, although well-differentiated tumors tended to have higher immune infiltration, which is in line with the current literature [33,42]. The anatomical site of SCCHN was unrelated to CPS, while higher TIL density was observed in OPSCC compared to other subsites. This comes as no surprise, due to the abundance of lymphoid tissue in the oropharyngeal region [13].

In terms of survival, the current research mainly intended to answer the foregoing research query: this led to selection bias in our patient population, as patients were not included in the study unconditionally, but according to the availability of tissue samples. We acknowledge this as a weakness of our study. Nonetheless, we found T stage to be the only routinely used clinicopathological variable that was associated with OS, but not with DFS. PD-L1 CPS showed no association with DFS outcome, and only a trend towards improved outcome for OS calculated from time of diagnosis. These results are controversial to previous publications in SCCHN, which report a survival benefit for PD-L1+ tumors, although none of these used the validated CPS scoring method [35,43,44,45,46]. Comparable to our study, Hirshoren et al. [45] investigated PD-L1 CPS in a small cohort of OSCC and laryngeal SCC patients, and reported no association in terms of OS and DFS according to different CPS categories. Surprisingly, PD-L1 CPS could be significantly associated with prolonged OS when calculated from time of tissue acquirement. Therefore, the prognostic relevance of PD-L1 CPS should always be carefully interpreted. TILs, on the other hand, are well known for being deregulated regarding number and functionality in SCCHN, while holding relevant prognostic information. When assessing TILs according to the IIBWG-constructed protocol, we observed that tumors with high TIL density (cut-off 20%) were correlated to significantly improved outcome. This was confirmed after multivariate analysis, in which high TIL count and low T stage were considered independent prognostic factors, superior to PD-L1 status. The combination of both parameters indicated that pT1-2 tumors displaying high TIL count had the best OS outcome compared to other phenotypes. Moreover, TIL density was demonstrated to be the sole discriminating factor to determine outcome regarding DFS.

This retrospective study has some limitations. Firstly, we did not account for treatment-related bias in paired samples. PD-L1 expression may be affected when exposed to anti-neoplastic therapies, such as radiotherapy and/or chemotherapy. This has been initially described in lesions collected post-therapy in non-squamous cell lung cancer [22]. To this end, patients in this cohort were not treated with immunotherapy, thus the current study cannot be acknowledged as a clinical validation study, as the predictive value of PD-L1 expression regarding ICI response was not assessed. Secondly, we did not consider whether metastatic lesions were collected synchronously or metachronously with primary tumors or other lesions. Thirdly, we were unable to assess the predictive implications of both biomarkers in this cohort, which is the main application of PD-L1 testing. Lastly, we are aware that this study can only report on the technical and analytical issues of CPS discordances. Whether our results are of true clinical relevance should be confirmed by correlating them with the overall response rate of SCCHN patients treated with pembrolizumab. Unfortunately, these data were not available for this study population.

## 5. Conclusions

In summary, this is the first study that assessed concordance of the standardized PD-L1 CPS assay and IIBWG TIL quantification method in different paired sample types. We noticed that CPS and TILs were clearly affected by intratumoral heterogeneity as paired biopsy–resection samples delivered poor concordance. Ultimately, treatment decisions may be different when testing different tissue samples, hereby withdrawing patients from potential therapy response or exposing them to harmful side-effects. Our data suggest that intertumoral and temporal heterogeneity may also be causing discordances in PD-L1 CPS status in different sample types. Therefore, re-biopsy for performing CPS may be justified to gain knowledge of the most recent PD-L1 status, especially when only inferior-quality tissue material is available [22].

Taking current guidelines and our results into account, one should be aware that the result of PD-L1 testing for a certain patient (and thus the eligibility of anti-PD-1/PD-L1 treatment) is not only determined by the tumor biology, but also in a high degree by several technical issues: (1) sample type; resection material of primary tumor and lymph nodes seem more often to be PD-L1+ than the paired biopsy material, and, to lesser extent, distant metastases, (2) if the tissue contains insufficient stroma, PD-L1 CPS positivity will not be reached in tumors with low PD-L1 expression in their cells, such as in SCCHN, (3) utilizing the most recent samples to overcome temporal heterogeneity, while considering prior treatments, and (4) technical requirements to guarantee high-quality IHC staining, such as exclusion of decalcified tumor tissue, aspirate cell blocks or paraffin blocks older than 3 years [23,24,25,28]. The fact that these factors can cause variation in PD-L1 status, and that it is not clear how this affects response rates, may explain to some extent why PD-L1 CPS has repeatedly been proven to lack robustness as a stand-alone predictive biomarker in tailoring treatment with ICI.

## Figures and Tables

**Table 1:** Clinicopathological parameters.

Age, Years (Median (Range))		62 (42–82)
Gender	Female	13 (16)
	Male	67 (84)
Smoking history	≥20 PY	63 (79)
	<20 PY	17 (21)
Alcohol abuse (≥30 U/week)	Yes	50 (63)
	No	30 (68)
Tumor site	Oral cavity	39 (49)
	Oropharynx	12 (15)
	Hypopharynx	13 (16)
	Larynx	16 (20)
T stage	1	24 (30)
	2	20 (25)
	3	18 (23)
	4	18 (23)
N stage	0	46 (58)
	1+	34 (42)
Prognostic stage	I	16 (20)
	II	12 (15)
	III	14 (18)
	IVa	32 (40)
	IVb	4 (5)
	IVc	2 (3)
Grade of differentiation	Good	7 (9)
	Moderate	42 (53)
	Poor	26 (33)
	Basaloid	5 (6)
Primary therapy	Surgery	56 (70)
	Radiotherapy	11 (14)
	Radiochemotherapy	12 (15)
	Chemotherapy	1 (1)

Data denote numbers (%), unless otherwise indicated. PY, pack-years.

**Table 2:** Tissue specimen characteristics.

Tissue Type	Blocks	TILs *	TILs ≥ 20%	CPS *	CPS < 1	CPS 1–19	CPS ≥ 20
Biopsy	58	15 (1–90)	24/53 ^§^	2 (0–60)	18	35	5
Resection	63	20 (0–80)	36	5 (0–100)	9	39	15
Lymph node	36	20 (0–80)	15/26 ^§^	8 (0–100)	5	22	9
Distant metastasis	6	4 (0–26)	1	3 (0–30)	3	3	0

Data denote numbers. * TILs and CPS are denoted as median (range). ^§^ Tissue samples that could not be evaluated because TILs were not included. Cut-off for TILs was determined post hoc for survival analysis. CPS, combined positive score; TIL, tumor-infiltrating lymphocyte.

**Table 3:** Association for dichotomous CPS and TILs between paired tissue material.

***A. Biopsy* vs. *resection***						
CPS		Biopsy				
		<1		≥1		*p*
Resection	<1	2	(14)	5	(17)	1.0000
	≥1	12	(86)	25	(83)	
TIL_s_		Biopsy				
		<20%		≥20%		*p*
Resection	<20%	14	(56)	3	(19)	0.0251
	≥20%	11	(44)	13	(81)	
***B. Biopsy* vs. *lymph node***						
CPS		Biopsy				
		<1		≥1		*p*
Lymph node	<1	3	(38)	0	(0)	0.0686
	≥1	5	(63)	10	(100)	
TIL_s_		Biopsy				
		<20%		≥20%		*p*
Lymph node	<20%	2	(50)	4	(40)	1.0000
	≥20%	2	(50)	6	(60)	
***C. Resection* vs. *lymph node***						
CPS		Resection				
		<1		≥1		*p*
Lymph node	<1	1	(14)	3	(17)	0.1481
	≥1	0	(86)	23	(83)	
TIL_s_		Resection				
		<20%		≥20%		*p*
Lymph node	<20%	3	(43)	5	(46)	1.0000
	≥20%	4	(57)	6	(54)	
***D. Primary tumor* vs. *metastasis***						
CPS		Primary tumors				
		<1		≥1		*p*
Metastasis	<1	2	(40)	4	(13)	0.1858
	≥1	3	(60)	27	(87)	
TIL_s_		Primary tumors				
		<20%		≥20%		*p*
Metastasis	<20%	5	(56)	7	(44)	0.6882
	≥20%	4	(44)	9	(56)	

Data denote numbers (% per column). *p* value for bicategorical variables was calculated using Fisher exact test, and for tricategorical variables using a chi-square test. CPS, combined positive score; TILs, tumor-infiltrating lymphocytes.

**Table 4:** Clinicopathological variables vs. CPS and TILs status.

Variables	CPS < 1		CPS 1–19		CPS ≥ 20		*p*	TILs < 20%		TILs ≥ 20%		*p*
Age < 65 years												
No	7	(14)	30	(61)	12	(24)	0.8614	22	(45)	27	(55)	0.7960
Yes	5	(16)	20	(65)	6	(19)		13	(42)	18	(58)	
Second primary												
No	7	(12)	37	(64)	14	(24)	0.4686	26	(45)	32	(55)	0.8051 *
Yes	5	(23)	13	(59)	4	(18)		9	(41)	13	(59)	
Smoker												
<20 PY	4	(24)	6	(35)	7	(41)	0.0310	9	(53)	8	(47)	0.4208 *
≥20 PY	8	(13)	44	(70)	11	(17)		26	(41)	37	(59)	
Alcohol abuse												
No	5	(17)	15	(50)	10	(33)	0.1501	9	(30)	21	(70)	0.0657 *
Yes	7	(14)	35	(70)	8	(16)		26	(52)	24	(48)	
P16 status												
Negative	10	(14)	45	(63)	16	(23)	0.8060	31	(44)	40	(56)	1.0000 *
Positive	2	(22)	5	(56)	2	(22)		4	(44)	5	(56)	
Topography												
Oral cavity	6	(15)	24	(62)	9	(23)	0.8115	14	(36)	25	(64)	0.1328
Oropharynx	1	(8)	9	(75)	2	(17)		4	(33)	8	(67)	
Hypopharynx	1	(8)	8	(62)	4	(31)		6	(46)	7	(54)	
Larynx	4	(25)	9	(56)	3	(19)		11	(69)	5	(31)	
Differentiation												
Good	3	(43)	4	(57)	0	(0)	0.0007	2	(29)	5	(71)	0.8106
Moderate	6	(14)	31	(74)	5	(12)		20	(48)	22	(52)	
Poor	1	(4)	12	(46)	13	(50)		11	(42)	15	(58)	
Basaloid	2	(40)	3	(60)	0	(0)		2	(40)	3	(60)	
AJCC stage												
I/II	4	(14)	19	(68)	5	(18)	0.7316	11	(39)	17	(61)	0.5572
III/IV	8	(15)	31	(60)	13	(25)		24	(46)	28	(54)	
T stage												
1–2	7	(16)	29	(66)	8	(18)	0.5927	19	(43)	25	(57)	0.9104
3–4	5	(14)	21	(58)	10	(28)		16	(44)	20	(56)	
N stage												
0	10	(22)	26	(57)	10	(22)	0.1406	19	(41)	27	(59)	0.6103
1+	2	(6)	24	(71)	8	(24)		16	(47)	18	(53)	

Data denote numbers (% per row). * *p* values determined via chi-square tests or via Fisher exact test. CPS, combined positive score; PY, pack-years; str, stromal; TILs, tumor-infiltrating lymphocytes.

## Data Availability

All data generated and analyzed during this study can be retrieved by sending a formal request by email to the corresponding author.

## Supplementary Materials

The following supporting information can be downloaded at: https://www.mdpi.com/article/10.3390/cancers14102431/s1. Figure S1: Comparison for CPS between paired tissue specimens, Figure S2: Comparison for CPS and TILs between patients for who biopsy, resection and lymph node material is present, Figure S3: Correlation for CPS between paired tissue specimens, Figure S4: Comparison for TIL count between paired tissue specimens, Figure S5: Correlation for TIL count between paired tissue specimens, Figure S6: Correlation between CPS and TILs, irrespective of tissue origin, Figure S7: Comparison for CPS and TIL count according to tumor differentiation and topography, Figure S8: Effect of CPS and TIL count on DFS in SCCHN patients, Figure S9: Histological sections of SCCHN depicting the heterogeneous profiles of PD-L1 staining and immune infiltration, Table S1: Paired analysis for CPS, Table S2: Correlation for CPS between paired tissue material, Table S3: Association for CPS between paired tissue material, Table S4: Association of CPS between primary tumor-based biopsy, primary tumor-based resection and lymph node material, Table S5: Paired analysis for TILs, Table S6: Correlation for TILs between paired tissue material, Table S7: Association of TILs between primary tumor-based biopsy, primary tumor-based resection and lymph node material, Table S8: Correlation between pathological parameters and time, Table S9: Correlation between CPS and TILs, Table S10: DFS analysis since time of tissue acquirement, Table S11: OS analysis since time of diagnosis, Table S12: OS analysis since time of tissue acquirement.
