# Supplementary material for: Concordance, Correlation, and Clinical Impact of Standardized PD-L1 and TIL Scoring in SCCHN
Source: Cancers (Basel). 2022 May 14;14(10):2431. doi: 10.3390/cancers14102431 (PMC9139955; doi:10.3390/cancers14102431)
Supplement: Supplementary file 1 [file cancers-14-02431-s001.zip › cancers-1704107-supplementary.pdf]

cancers

MDPI

Concordance, Correlation, and Clinical Impact of Standardized PD-L1 and TIL Scoring in SCCHN

Stijn Jeroen De Keukeleire [1,2,*], Tijl Vermassen [1,3,4], Philippe Deron [5], Wouter Huvenne [3,5], Fréderic Duprez [6], David Creytens [4,7], Jo Van Dorpe [7], Liesbeth Ferdinande [7] and Sylvie Rottey [1,3,4]

[1] Department of Medical Oncology, University Hospital Ghent, 9000 Ghent, Belgium; tijl.vermassen@uzgent.be (T.V.); sylvie.rottey@ugent.be (S.R.)
[2] Department of Internal Medicine, University Hospital Brussels, 1090 Jette, Belgium
[3] Drug Research Unit Ghent, Ghent University Hospital, 9000 Ghent, Belgium
[4] Cancer Research Institute Ghent (CRIG), 9000 Ghent, Belgium
[5] Department of Head and Neck Surgery, Ghent University Hospital, 9000 Ghent, Belgium; philippe.deron@uzgent.be (P.D.); wouter.huvenne@uzgent.be (W.H.)
[6] Department of Radiation Oncology, Ghent University Hospital, 9000 Ghent, Belgium; frederic.duprez@uzgent.be
[7] Department of Pathology, Ghent University Hospital, 9000 Ghent, Belgium; david.creytens@uzgent.be (D.C.); jo.vandorpe@uzgent.be (J.V.D.); liesbeth.ferdinande@ugent.be (L.F.)
* **Correspondence:** stijn.dekeukeleire@uzbrussel.be; Tel.: +32-2-477-41-11

Table S1. Paired analysis for CPS

Tissue	N	Median (range)	*P*
Biopsy ↔ resection	44	2 (0–60) ↔ 5 (0–70)	0.0085
Resection ↔ lymph node	27	5 (0–100) ↔ 8 (0–100)	0.0495
Biopsy ↔ lymph node	18	1 (0–15) ↔ 8 (0–45)	0.0076
Primary tumor ↔ metastasis	36	5 (0–100) ↔ 8 (0–100)	0.3669
Biopsy ↔ resection ↔ lymph node	11	1 (0–10) ↔ 5 (1–30) ↔ 5 (0–20)	0.0017 *

* significant difference for biopsy versus resection and for biopsy versus lymph node. CPS, combined positive score.

Table S2. Correlation for CPS between paired tissue material

Correlation	n	r	*P*
Biopsy versus resection	44	0.3537	0.0185
Resection versus lymph node	27	0.8592	<0.0001
Biopsy versus lymph node	18	−0.0281	0.9119
Primary tumor versus metastasis	36	0.7512	<0.0001

CPS, combined positive score.

Table S3. Association for CPS between paired tissue material

A. Biopsy versus resection

		Biopsy			
		<1	1–19	≥20	***P***
**Resection**	<1	2 (14)	5 (19)	0 (0)	0.1986
	1–19	11 (79)	16 (59)	1 (33)	
	≥20	1 (7)	6 (22)	2 (67)	

B. Biopsy versus lymph node

		Biopsy			
		<1	1–19	≥20	***P***
**Lymph node**	<1	3 (38)	0 (0)	0 (0)	0.1054
	1–19	4 (50)	8 (80)	0 (0)	
	≥20	1 (13)	2 (20)	0 (0)	

C. Resection versus lymph node

		Resection			
		<1	1–19	≥20	***P***
**Lymph node**	<1	1 (100)	3 (18)	0 (0)	0.0016
	1–19	0 (0)	13 (77)	3 (33)	
	≥20	0 (0)	1 (6)	6 (67)	

D. Primary tumor versus metastasis

		Primary tumors			
		<1	1–19	≥20	***P***
**Metastasis**	<1	2 (40)	0 (0)	4 (18)	0.0082
	1–19	1 (20)	6 (67)	2 (9)	
	≥20	2 (40)	3 (33)	16 (73)	

Data denote numbers (% per column). *P* value for bicategorical variables calculated using Fisher Exact test, for tricategorical variables using Chi Square test. CPS, combined positive score.

Table S4. Association of CPS between primary tumor-based biopsy, primary tumor-based resection and lymph node material

Case	*Biopsy*		*Resection*		*Lymph node*	
	Continuous	Categorical.	Continuous	Categorical.	Continuous	Categorical.
1	0	<1	5	1–19	0	<1
2	0	<1	5	1–19	0	<1
3	0	<1	1	1–19	2	1–19
4	0	<1	4	1–19	5	1–19
5	0	<1	5	1–19	8	1–19
6	**1**	**1–19**	**10**	**1–19**	**8**	**1–19**
7	**2**	**1–19**	**2**	**1–19**	**5**	**1–19**
8	2	1–19	25	≥20	8	1–19
9	**3**	**1–19**	**3**	**1–19**	**2**	**1–19**
10	4	1–19	30	≥20	20	≥20
11	10	1–19	28	≥20	15	1–19

Samples with perfect concordance according to categorical CPS are indicated in green. Perfect concordance was found in 3/11 (27%) cases. CPS, combined positive score.

Table S5. Paired analysis for TIL_{str}

**Tissue**	**N**	**Median (range)**	***P***
Biopsy ↔ resection	41	15 (1–90) ↔ 20 (0–80)	0.2363
Resection ↔ lymph node	18	20 (1–80) ↔ 20 (0–65)	0.8871
Biopsy ↔ lymph node	14	30 (5–90) ↔ 20 (0–50)	0.1167
Primary tumor ↔ metastasis	25	20 (0–80) ↔ 20 (1–65)	0.6263
Biopsy ↔ resection ↔ lymph node	8	30 (5–90) ↔ 20 (5–80) ↔ 18 (0–50)	0.4630

str, stromal; TIL, tumor infiltrating lymphocyte.

Table S6. Correlation for TIL_{str} between paired tissue material

**Correlation**	***n***	**r**	***P***
Biopsy versus resection	41	0.4807	0.0015
Resection versus lymph node	18	0.1394	0.5811
Biopsy versus lymph node	14	0.0713	0.8087
Primary tumor versus metastasis	25	0.1671	0.4247

str, stromal; TIL, tumor infiltrating lymphocyte.

Table S7. Association of TIL_{str} between primary tumor-based biopsy, primary tumor-based resection and lymph node material

Case	*Biopsy*		*Resection*		*Lymph node*	
	Continuous	Categorical.	Continuous	Categorical.	Continuous	Categorical.
1	15	<20	40	≥20	20	≥20
2	20	≥20	20	≥20	10	<20
3	**10**	**<20**	**5**	**<20**	**4**	**<20**
4	**–**	**–**	**20**	**≥20**	**65**	**≥20**
5	5	<20	20	≥20	20	≥20
6	18	<20	50	≥20	–	–
7	**40**	**≥20**	**20**	**≥20**	**50**	**≥20**
8	60	≥20	50	≥20	15	<20
9	**20**	**≥20**	**–**	**–**	**20**	**≥20**
10	15	<20	70	≥20	–	–
11	60	≥20	8	<20	50	≥20

Samples with perfect concordance according categorical TIL_{str} are indicated in green. Perfect concordance was found in 4/11 (36%) cases. Of note, TILs could not be determined for 4 cases (one biopsy, one resection and two lymph nodes). str, stromal; TIL, tumor infiltrating lymphocyte.

Table S8. Correlation between pathological parameters and time

**Samples**	**n**	**r**	***P* value**
A. ΔCPS – Δtime			
All samples	56	−0.0784	0.5658
Biopt versus resection	44	0.1820	0.2370
Biopt versus lymph node	18	0.1217	0.6305
Resection versus lymph node	9	0.0687	0.8605
B. ΔCPS – log(Δtime)			
All samples	56	−0.0758	0.5789
Biopt versus resection	44	0.1492	0.3339
Biopt versus lymph node	18	0.1778	0.4804
Resection versus lymph node	9	−0.0492	0.9000
C. $ΔTIL_{str}$ – Δtime			
All samples	53	−0.1646	0.2389
Biopt versus resection	41	0.1505	0.3476
Biopt versus lymph node	13	−0.1479	0.6296
Resection versus lymph node	8	-0.0919	0.8287
D. $ΔTIL_{str}$ – log(Δtime)			
All samples	53	−0.1428	0.3076
Biopt versus resection	41	0.1167	0.4676
Biopt versus lymph node	13	−0.1463	0.6335
Resection versus lymph node	8	−0.0444	0.9169

ΔCPS, ΔTILs and Δtime were calculated as the difference in CPS, in TILs and in time, respectively, between the second and the first specimen type. For 'all samples', the specimens with the longest Δtime were selected. CPS, combined positive score; str, stromal; TIL, tumor infiltrating lymphocyte.

**Table S9. Correlation between CPS and TIL_{str}**			
**Correlation**	***n***	**r**	***P***
A. All samples			
CPS versus TIL_{str}	146	0.2067	0.0123
CPS versus log (TIL_{str})	144	0.2151	0.0096
Log (CPS) versus TIL_{str}	116	0.2274	0.0141
Log (CPS) versus log (TIL_{str})	116	0.2692	0.0035
B. Biopsy			
CPS versus TIL_{str}	53	0.0550	0.6959
CPS versus log (TIL_{str})	53	0.0853	0.5438
Log (CPS) versus TIL_{str}	39	0.1799	0.2732
Log (CPS) versus log (TIL_{str})	39	0.1835	0.2634
C. Resection			
CPS versus TIL_{str}	62	0.2680	0.0352
CPS versus log (TIL_{str})	61	0.2331	0.0707
Log (CPS) versus TIL_{str}	53	0.2508	0.0701
Log (CPS) versus log (TIL_{str})	53	0.2698	0.0507
D. Lymph node			
CPS versus TIL_{str}	25	0.1584	0.4494
CPS versus log (TIL_{str})	25	0.2338	0.2607
Log (CPS) versus TIL_{str}	21	0.2291	0.3177
Log (CPS) versus log (TIL_{str})	21	0.3983	0.0737
E. Distant metastasis			
CPS versus TIL_{str}	6	0.9757	0.0009
CPS versus log (TIL_{str})	5	0.8293	0.0825
Log (CPS) versus TIL_{str}	3	0.9737	0.1464
Log (CPS) versus log (TIL_{str})	3	0.9081	0.2750
F. Samples selected for survival analysis			
CPS versus TIL_{str}	80	0.2332	0.0373
CPS versus log (TIL_{str})	77	0.2134	0.0623
Log (CPS) versus TIL_{str}	68	0.2396	0.0491
Log (CPS) versus log (TIL_{str})	68	0.2870	0.0177

For log (TIL_{str}), all cases with TILs = 0% were excluded. For log (CPS), all cases with CPS = 0 were excluded. For log (TIL_{str}) and log (CPS), all cases with no TIL_{str} = 0% and / or CPS = 0 were excluded. CPS, combined positive score; str, stromal; TIL, tumor infiltrating lymphocyte.

Table S10. DFS analysis since time of tissue acquirement

Parameter	Univariate analysis		
	Median DFS (95% CI)	HR (95% CI)	*P*
**T stage**			
1–2	3.7 (1.5–4.0)	1	
3–4	1.6 (0.9–4.9)	1.42 (0.78–2.59)	0.2520
**N stage**			
0	2.4 (1.4–4.0)	1	
1+	3.7 (1.2–4.9)	0.78 (0.43–1.42)	0.4212
**AJCC stage**			
I–II	2.8 (1.0–3.4)	1	
III–IV	2.7 (1.4–4.9)	1.15 (0.62–2.12)	0.6562
**Alcohol abuse (≥30 U / week)**			
No	2.7 (1.0–4.9)	1	
Yes	2.4 (1.3–3.7)	1.21 (0.66–2.24)	0.5335
**Smoking history**			
<20 PY	3.4 (0.8–3.4)	1	
≥20 PY	2.6 (1.3–4.0)	1.34 (0.66–2.70)	0.4141
**TIL_{str}**			
<20%	1.4 (0.8–2.3)	1	
≥20%	4.0 (2.6–4.9)	0.45 (0.24–0.84)	0.0126
**CPS**			
<1	1.5 (0.1–3.4)	1	
≥1	2.7 (1.5–4.9)	0.68 (0.78–1.70)	0.4070

Parameters that reached significance on univariate analysis, were included in the multivariate analysis. CI, confidence interval; CPS, combined positive score; DFS, disease-free survival; HR, hazard ratio; PY, pack-years; str, stromal; TIL, tumor infiltrating lymphocyte.

Table S11. OS analysis since time of diagnosis

Parameter	Univariate analysis			Multivariate analysis	
	Median OS (95% CI)	HR (95% CI)	*P*	HR (95% CI)	*P*
**T stage**					
1–2	NR	1		1	
3–4	3.8 (1.9–5.0)	2.12 (1.07–4.18)	0.0310	2.06 (1.05–4.03)	0.0351
**N stage**					
0	NR	1			
1+	4.4 (2.4–5.0)	1.09 (0.55–2.14)	0.8055		
**AJCC stage**					
I–II	NR	1			
III–IV	4.2 (2.6–5.0)	1.73 (0.88–3.43)	0.1148		
**Alcohol abuse (≥30 U / week)**					
No	5.0 (3.9–5.0)	1			
Yes	3.8 (2.4–4.4)	1.82 (0.92–3.61)	0.0867		
**Smoking history**					
<20 PY	NR	1			
≥20 PY	4.2 (2.6–5.0)	2.00 (0.92–4.35)	0.0815		
**TIL_{str}**					
<20%	2.4 (1.6–4.4)	1		1	
≥20%	5.0 (3.9–5.0)	0.41 (0.21–0.80)	0.0098	0.42 (0.21–0.83)	0.0123
**CPS**					
<1	1.4 (0.4–1.6)	1			
≥1	5.0 (3.8–5.0)	0.33 (0.11–1.01)	0.0516		

Parameters that reached significance on univariate analysis, were included in the multivariate analysis. CI, confidence interval; CPS, combined positive score; HR, hazard ratio; OS, overall survival; PY, pack-years; str, stromal; TIL, tumor infiltrating lymphocyte.

Table S12. OS analysis since time of tissue acquirement

Parameter	Univariate analysis			Multivariate analysis	
	Median OS (95% CI)	HR (95% CI)	*P*	HR (95% CI)	*P*
**T stage**					
1–2	NR	1		1	
3–4	3.3 (1.5–4.9)	2.07 (1.05–4.09)	0.0363	2.17 (1.11–4.27)	0.0241
**N stage**					
0	4.1 (2.6–4.1)	1			
1+	4.9 (1.8–4.9)	1.06 (0.54–2.09)	0.8589		
**AJCC stage**					
I–II	NR	1			
III–IV	3.7 (1.9–4.9)	1.63(0.82–3.28)	0.1664		
**Alcohol abuse (≥30 U / week)**					
No	4.9 (3.7–4.9)	1			
Yes	3.3 (1.8–3.3)	1.92 (0.97–3.80)	0.0603		
**Smoking history**					
<20 PY	NR	1			
≥20 PY	3.7 (2.4–4.9)	2.02 (0.93–4.42)	0.0766		
**TIL_{str}**					
<20%	1.7 (1.3–2.4)	1		1	
≥20%	4.9 (3.7–4.9)	0.36 (0.20–0.72)	0.0038	0.36 (0.18–0.71)	0.0033
**CPS**					
<1	1.4 (0.1–1.5)	1		1	
≥1	4.9 (3.6–4.9)	0.31 (0.10–0.95)	0.0406	0.49 (0.21–1.15)	0.1000

Parameters that reached significance on univariate analysis, were included in the multivariate analysis. CI, confidence interval; CPS, combined positive score; HR, hazard ratio; OS, overall survival; PY, pack-years; str, stromal; TIL, tumor infiltrating lymphocyte.

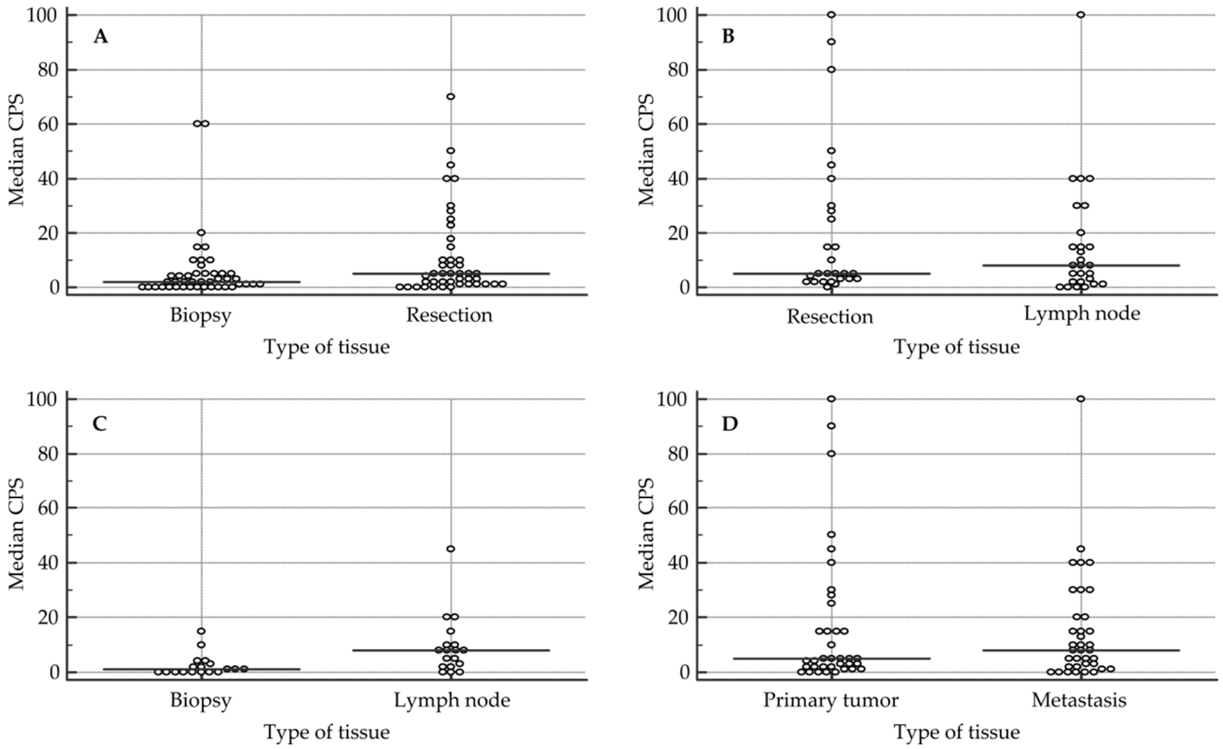

Figure S1. Comparison for CPS between paired tissue specimens. Median CPS is indicated in the Y-axis, type of tissue in the X-axis. Comparisons are illustrated for (A) biopsy versus resection material (P = 0.0085), (B) resection versus lymph node material (P = 0.0495), (C) biopsy versus lymph node material (P = 0.0076), and (D) primary tumor versus metastatic material (P = 0.3669). CPS, combined positive score.

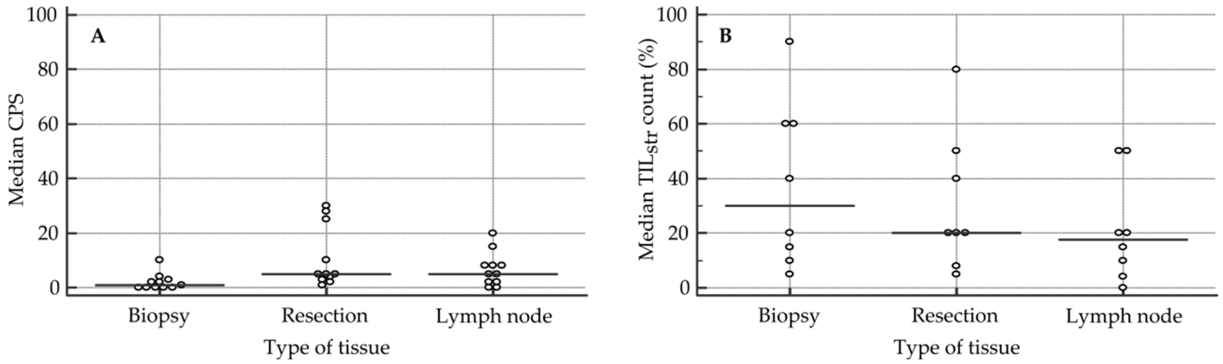

Figure S2. Comparison for CPS and TILstr between patients for who biopsy, resection and lymph node material is present. Median CPS or TIL_{str} count is indicated in the Y-axis, type of tissue in the X-axis. Comparisons are illustrated for (A) median CPS (P = 0.0017), (B) TIL_{str} count (P = 0.4630). CPS, combined positive score; str, stromal, TIL, tumor infiltrating lymphocyte.

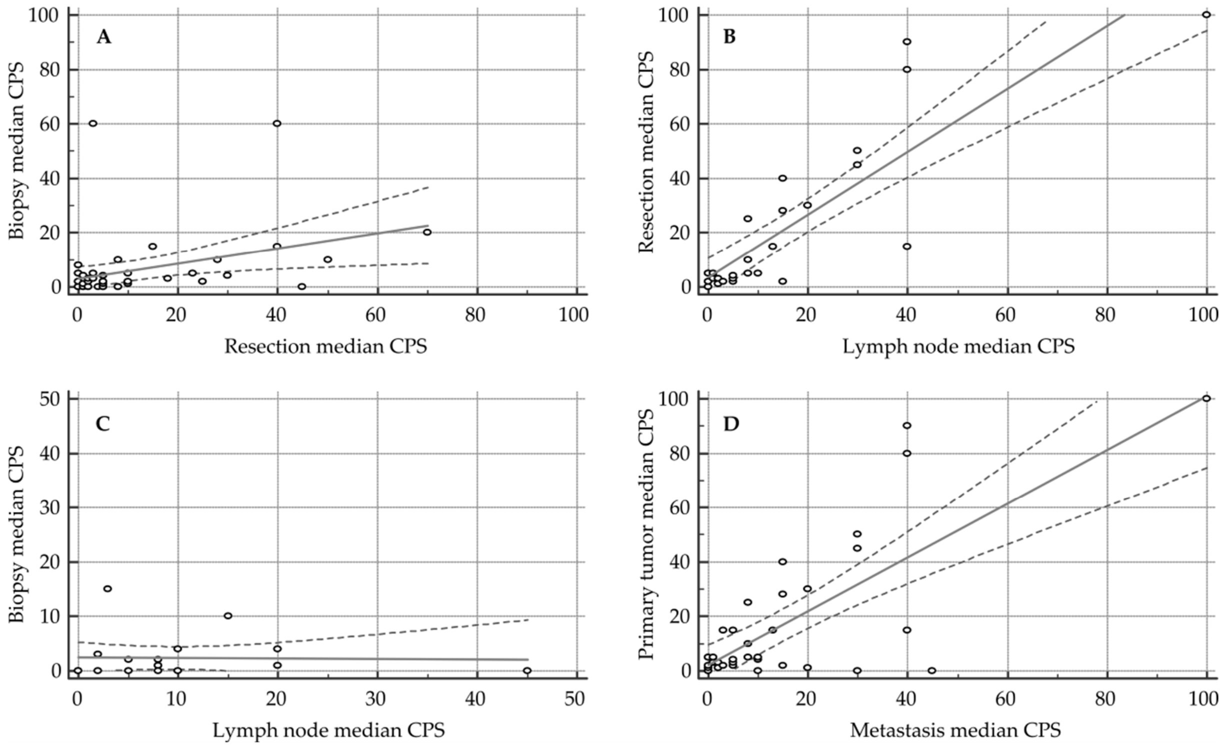

Figure S3. Correlation for CPS between paired tissue specimens. Correlations are illustrated for (A) biopsy versus resection material (r = 0.3537; P = 0.0185), (B) resection versus lymph node material (r = 0.8592; P < 0.0001), (C) biopsy versus lymph node material (r =−0.0281; P = 0.9119), and (D) primary tumor versus metastatic material (r = 0.7512; P < 0.0001). CPS, combined positive score.

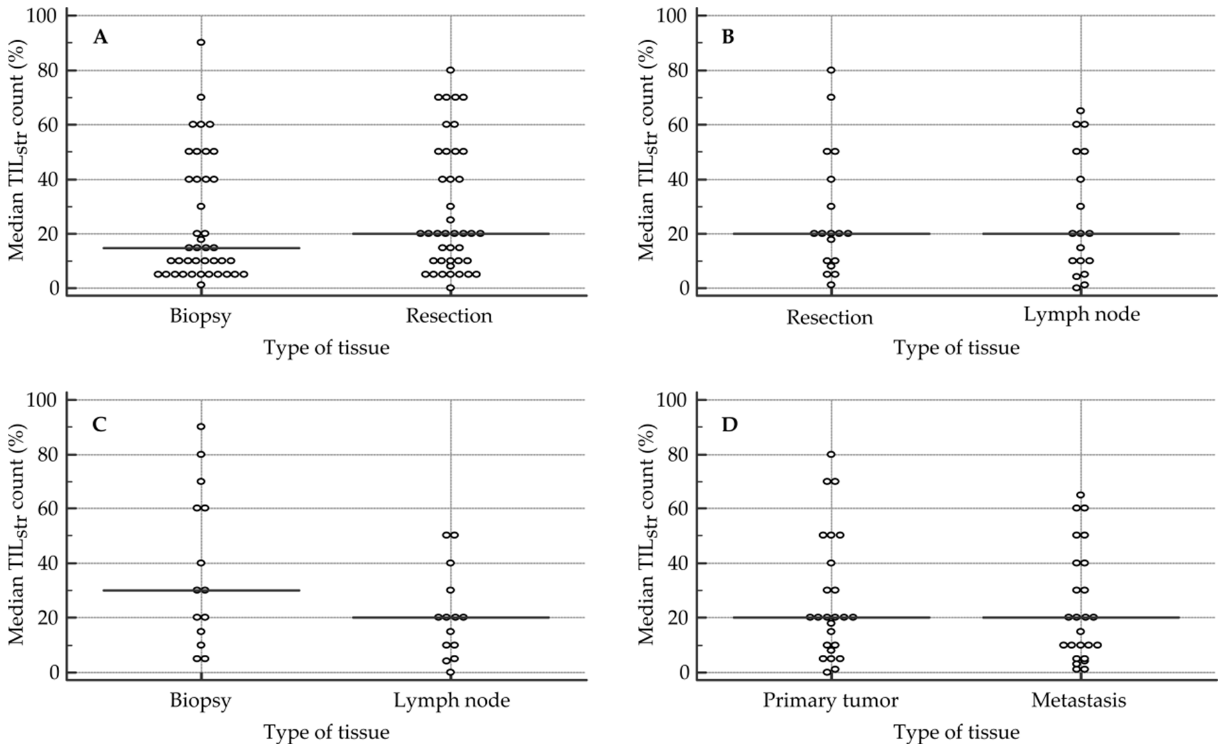

Figure S4. Comparison for TIL_{str} count between paired tissue specimens. Median TIL_{str} count is indicated in the Y-axis, type of tissue in the X-axis. Comparisons are illustrated for (A) biopsy versus resection material (P = 0.2363), (B) resection versus lymph node material (P = 0.8871), (C) biopsy versus lymph node material (P = 0.1167), and (D) primary tumor versus metastatic material (P = 0.6263). str, stromal, TIL, tumor infiltrating lymphocyte.

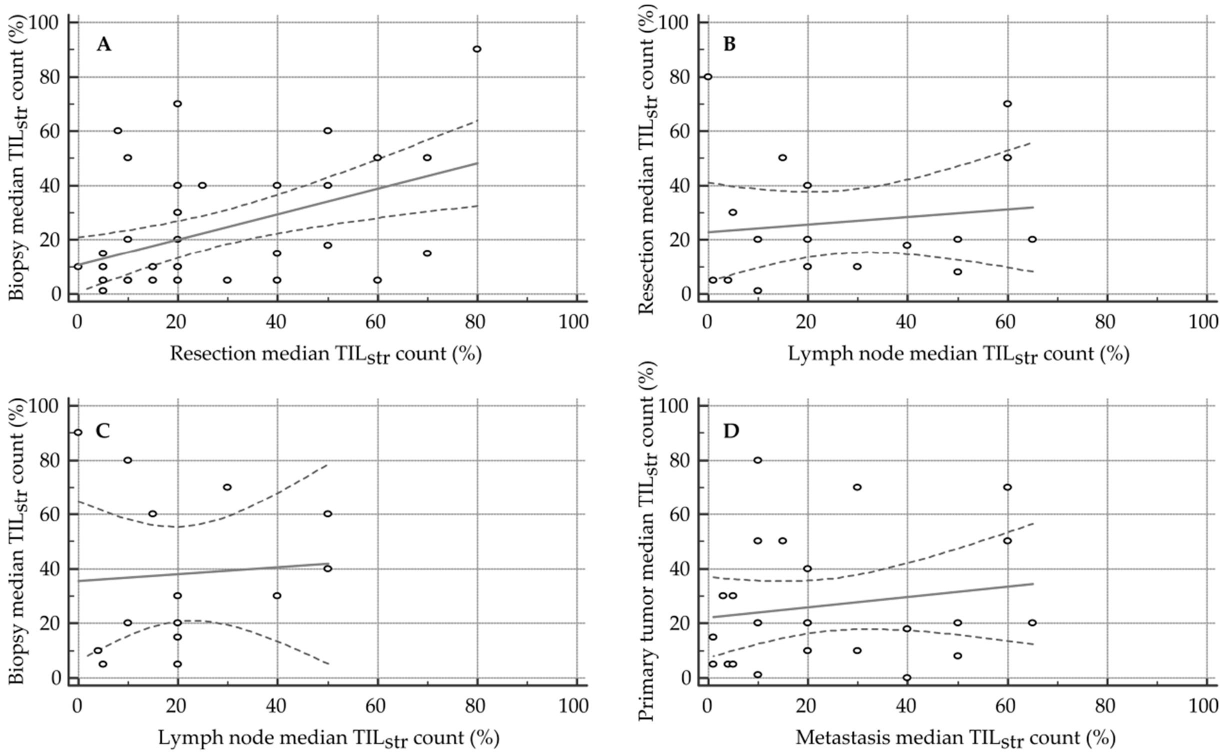

Figure S5. Correlation for TIL_{str} between paired tissue specimens. Correlations are illustrated for (A) biopsy versus resection material (r = 0.4807; P = 0.0015), (B) resection versus lymph node material (r = 0.1394; P = 0.5811), (C) biopsy versus lymph node material (r = 0.0713; P = 0.8087), and (D) primary tumor versus metastatic material (r = 0.1671; P = 0.4247). str, stromal; TIL, tumor infiltrating lymphocyte.

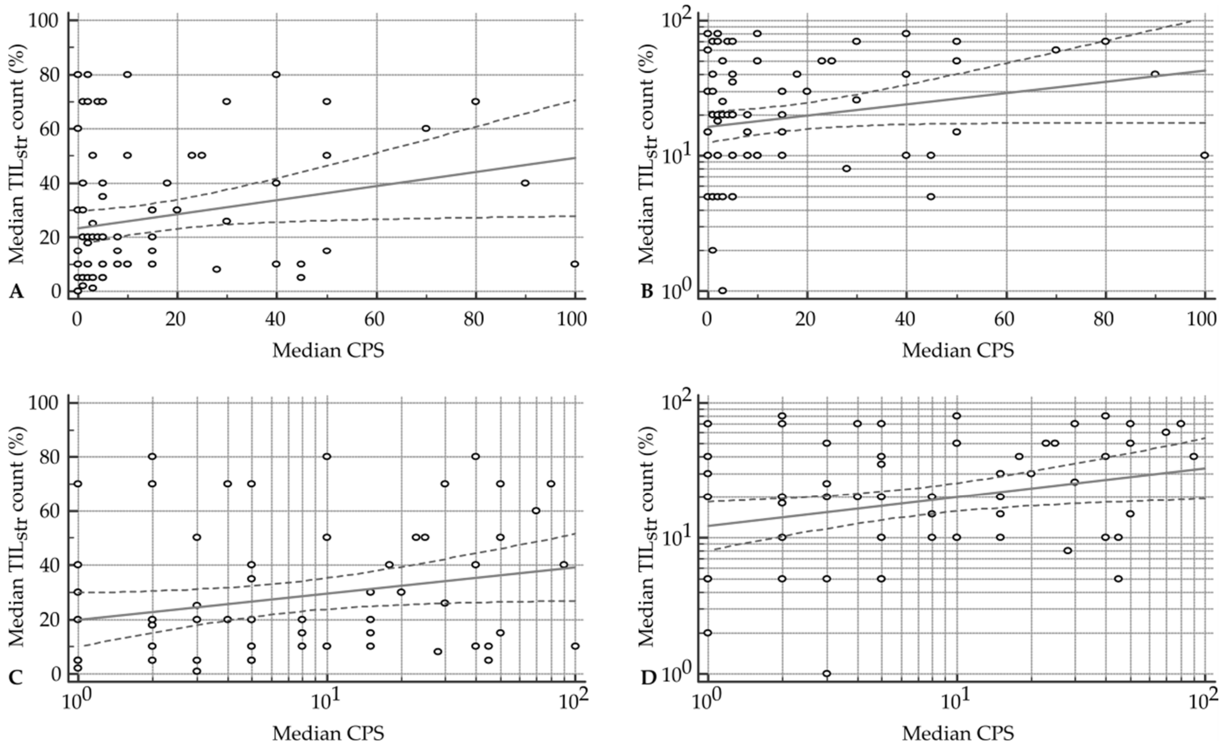

Figure S6. Correlation between CPS and TIL_{str}, irrespective of tissue origin. Correlations are illustrated for (A) CPS versus TIL_{str} (r = 0.4807; P = 0.0015), (B) CPS versus log (TIL_{str}) (r = 0.1394; P = 0.5811), (C) log (CPS) versus TIL_{str} (r = 0.0713; P = 0.8087), and (D) log (CPS) versus log (TIL_{str}) (r = 0.1671; P = 0.4247). CPS, combined positive score; str, stromal; TIL, tumor infiltrating lymphocyte.

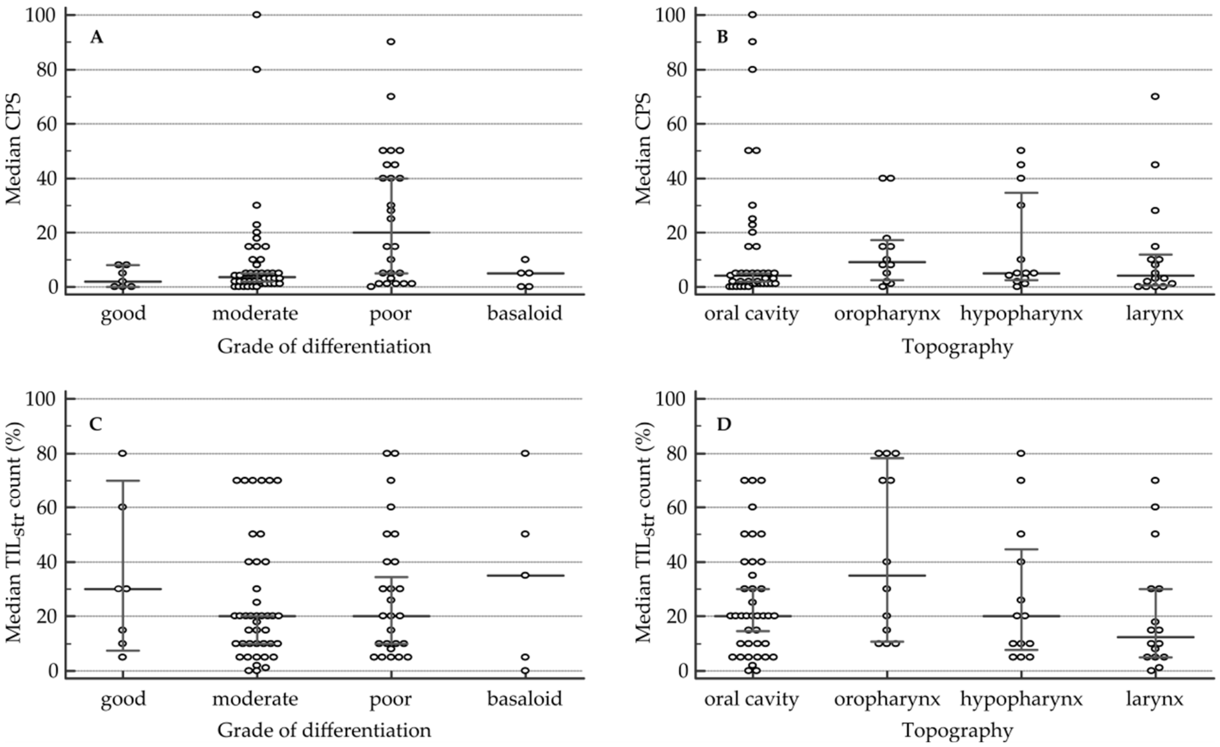

Figure S7. Comparison for CPS and TIL$_{str}$ count according to tumor differentiation and topography. Median CPS / TIL$_{str}$ count is indicated in the Y-axis, tumor differentiation / topography in the X-axis. Comparison are illustrated for (A) CPS according to tumor differentiation (*P* = 0.0101), (B) CPS according to topography (*P* = 0.5130), (C) TIL$_{str}$ count according to tumor differentiation (*P* = 0.8128), and (D) TIL$_{str}$ count according to topography (*P* = 0.1234). CPS, combined positive score; str, stromal, TIL, tumor infiltrating lymphocyte.

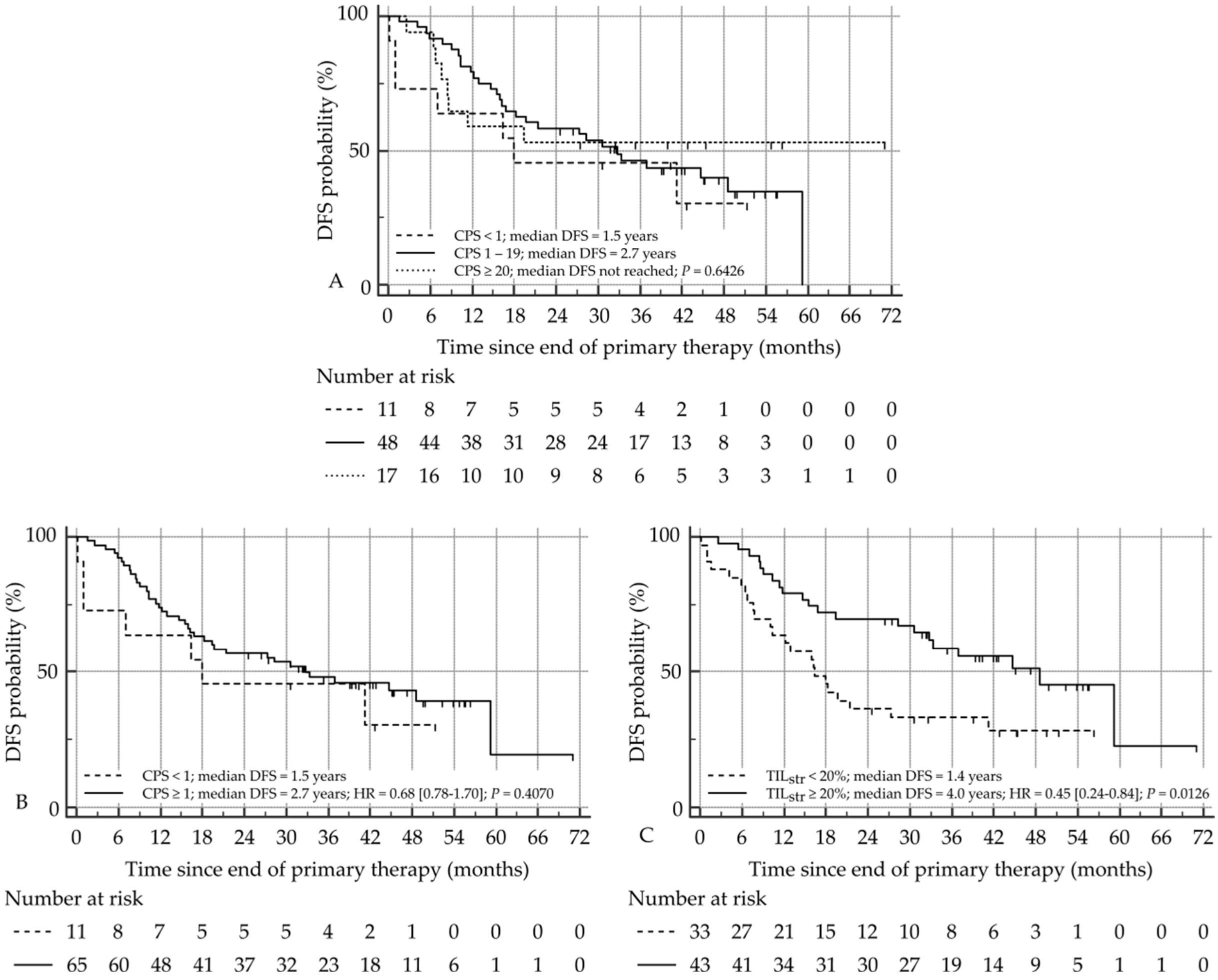

Figure S8. Effect of CPS and TIL_{str} count on DFS in SCCHN patients. X-axis depicts survival time (in months), Y-axis depicts the cumulative DFS probability. DFS has been depicted for (A) trichotomous CPS, (B) dichotomous CPS, and (C) TIL_{str}. Number at risk for each group has been indicated beneath each DFS curve. CPS, combined positive score; DFS, disease-free survival; HR, hazard ratio; SCCHN, squamous cell carcinoma of the head and neck; str, stromal; TIL, tumor infiltrating lymphocyte.

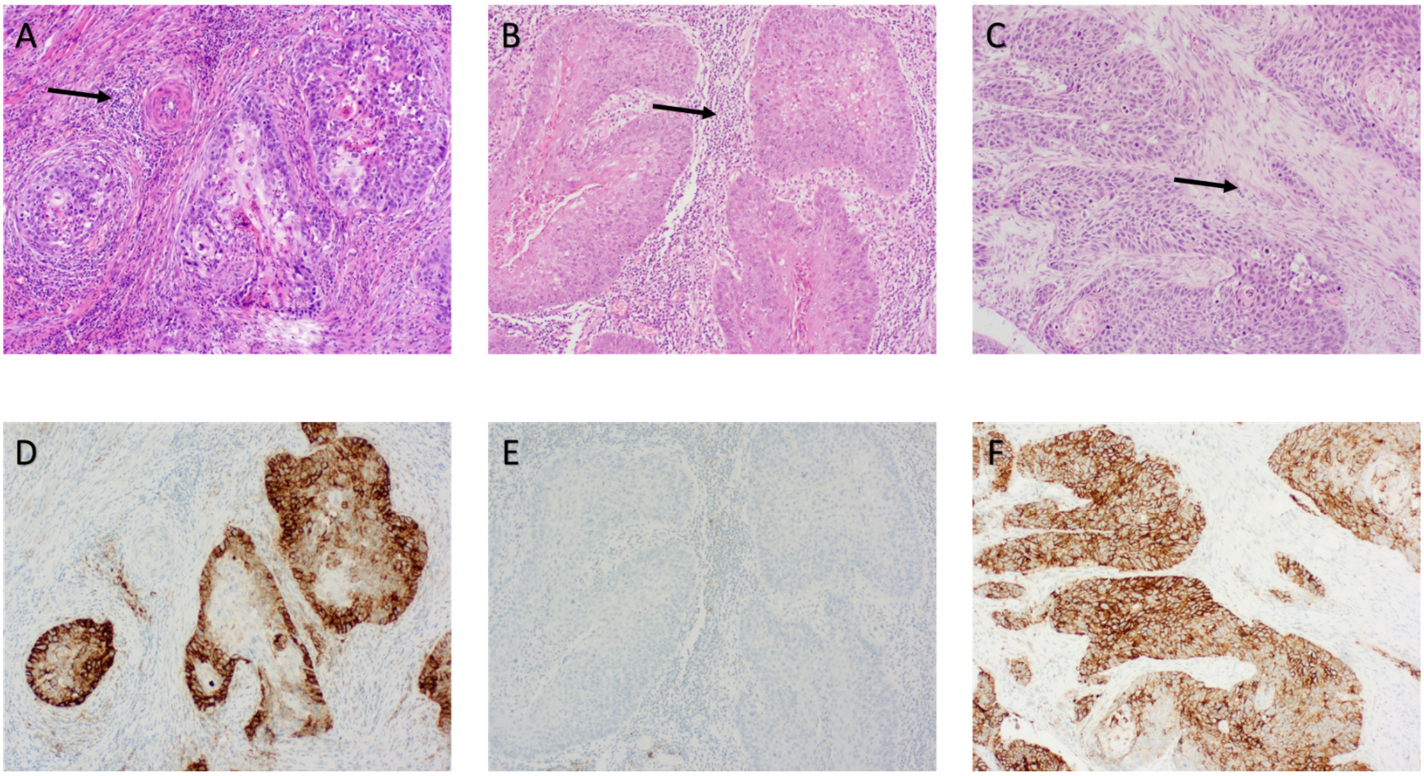

Figure S9. Histological sections of SCCHN depicting the heterogeneous profiles of PD-L1 staining and immune infiltration. The upper and lower row indicate paired HE and PD-L1 IHC slides, respectively. Profiles are shown for (1) high TIL_{str} (A) and high PD-L1 (D), (2) high TIL_{str} (B) and low PD-L1 (E), (3) low TIL_{str} (C) and high PD-L1 (F). Arrows: compartment with tumoral stroma. HE, haematoxilin-eosin; IHC, immunohistochemistry; PD-L1, programmed cell death-ligand 1; SCCHN, squamous cell cancer in head and neck; str, stromal; TIL, tumor infiltrating lymphocyte.
